# Tongues on the EDGE: language preservation priorities based on threat and lexical distinctiveness

**DOI:** 10.1098/rsos.171218

**Published:** 2017-12-13

**Authors:** Nicolas Perrault, Maxwell J. Farrell, T. Jonathan Davies

**Affiliations:** 1 University of Oxford, School of Archaeology, Oxford, UK; 2 McGill University, Department of Biology, Montréal, Québec, Canada; 3 African Centre for DNA Barcoding, Department of Botany and Plant Biotechnology, University of Johannesburg, Johannesburg, South Africa

**Keywords:** conservation, language preservation, biodiversity, phylogeny, evolutionarily distinct and globally endangered, linguistic diversity

## Abstract

Languages are being lost at rates exceeding the global loss of biodiversity. With the extinction of a language we lose irreplaceable dimensions of culture and the insight it provides on human history and the evolution of linguistic diversity. When setting conservation goals, biologists give higher priority to species likely to go extinct. Recent methods now integrate information on species evolutionary relationships to prioritize the conservation of those with a few close relatives. Advances in the construction of language trees allow us to use these methods to develop language preservation priorities that minimize loss of linguistic diversity. The evolutionarily distinct and globally endangered (EDGE) metric, used in conservation biology, accounts for a species’ originality (evolutionary distinctiveness—ED) and its likelihood of extinction (global endangerment—GE). Here, we use a similar framework to inform priorities for language preservation by generating rankings for 350 Austronesian languages. Kavalan, Tanibili, Waropen and Sengseng obtained the highest EDGE scores, while Xârâcùù (Canala), Nengone and Palauan are among the most linguistically distinct, but are not currently threatened. We further provide a way of dealing with incomplete trees, a common issue for both species and language trees.

## 1. Introduction

There is growing evidence that we are in the midst of a sixth mass extinction event and mankind is probably the cause [1,2]. Since the 1950s, scientific and public awareness of the loss of biodiversity has increased considerably [3], but we lack both resources and time to save all endangered species. Some species will go extinct and we must make choices and set priorities in species conservation [4]. Many human languages are equally, if not more threatened [5]. It is estimated that one of the world’s 7000 languages vanishes every other week and half might not survive the twenty-first century [6]. Languages are the spark of a people, the bearing of cultures, and are tied to a special understanding of native environments. Their disappearance is a loss to humanity, scholarship and science [7]. Prehistorians study languages to trace back population movements [8,9] and anthropologists use language trees to test hypothesis of cultural evolution [10,11]. Linguists use the variety of parlances to understand language as a human phenomenon; every single tongue gives them additional insight [7]. Traditional ecological knowledge, often used in biodiversity conservation efforts [12–15], is imperilled if languages are lost [16,17]. The rapid rate of language loss coupled with limited resources for preservation indicates that formal prioritization schemes may be useful tools to maximizing the retention of linguistic diversity.

In conservation biology, there have been efforts to prioritize species based on their evolutionary distinctiveness (ED) with the idea that highly distinct species might have unique traits that contribute to biodiversity [18–20] and that communities that capture greater phylogenetic diversity may enhance ecosystem functioning (e.g. [21,22]). For example, species with many close relatives might provide few unique ecosystem services. Conversely, species with few relatives are usually the most functionally original [20] and may thus provide irreplaceable services (see arguments in [23]). Likewise in linguistics, the more isolated a language is in its family tree, the more unique information it contains and ultimately contributes to linguistic diversity. Prioritizing the documentation of threatened and isolated languages is a key goal in linguistics [6]. Recently developed methods for quantifying similarity among languages [24] offer new opportunities to inform these prioritizations.

In biology, phylogenetic trees (trees of life) depict species ancestor-to-descendant relationships. Two populations of a single species will evolve into two species when gene flow is interrupted, often by geographical isolation [25]. One can consider speciation complete when two populations can no longer interbreed [26]. Speciation is depicted in the tree by the splitting of branches. Likewise, though a simplification, dialects become languages when the speakers of one dialect can no longer understand speakers of the other. Like new species, diverged dialects are splits in a language tree [27].

We can quantify a species’ ED by measuring how isolated it is on a phylogenetic tree. Species isolated in the tree are said to be evolutionarily distinct. Similarly, we can quantify linguistic distinctiveness from language trees. Once a set of features is selected and a tree built from them, distinctiveness scores can be calculated and used as empirical and objective estimates of uniqueness among languages. There are many distinctiveness metrics [28,29], but all aim to favour species with a few close relatives.

Early distinctiveness metrics counted only the number of splits in a species’ ancestry, giving higher scores to fewer splits [4,30]. Such metrics are highly sensitive to missing data (absent splits in the tree). More recent measures treat the lengths of tree branches as units of distinctiveness, usually counted in millions of years. In these cases, a species’ distinctiveness is equal to the length of its branch plus a fraction of that of its ancestors. Like money that people inherit from their mother, fewer siblings mean a larger inheritance. If the mother herself had few siblings, she inherited more from her parents and in turn would have more to leave to her children. Further, with a constant salary, the longer she lived, the more money she would have to leave them. Devised by Redding [31] and employed by Isaac *et al.* [18], we used a metric of ED in which ancestral distinctiveness is divided evenly among all living descendants, although distinctiveness may be calculated in other ways [29,32].

Isaac *et al.* [18] determined the ED from a near-complete species-level phylogenetic tree for mammals with branch lengths proportional to time. Implicit within their calculation is an assumption that species differentiate at a constant rate through time, i.e. that branch lengths measured in evolutionary time capture the expected differences between species. ED, being a weighted sum of branch lengths, also represents time in millions of species-years. The platypus, for example, has an ED of approximately 97.6 million years, the greatest ED in the mammal phylogeny.

The assumption of constant divergence through time, however, does not hold for languages. As Icelandic and Norwegian diverged from Old Norse one thousand years ago, the basic vocabulary of Norwegian has changed five times faster than that of Icelandic [33]. This is not an isolated example—time is a poor estimator of linguistic distinctiveness. A language’s ED is better computed from a language tree whose branch lengths convey distinctiveness directly. Here, we use a tree based on the proportion of ancestral words substituted for newer words in a language’s basic vocabulary.

To prioritize conservation efforts so as to minimize the expected loss of diversity, distinctiveness can be weighted by the probability of extinction—P(extinction) [34]. To estimate this probability, Isaac *et al.* [18] used the endangerment levels of the IUCN Red List [35], an objective qualitative scale of species extinction risk, assuming each increase in Red List threat category represents a doubling in P(extinction). Taking a species’ ED and global endangerment (GE) as proxies for its contribution to diversity and probability of extinction, a species’ EDGE score is calculated as follows:

1.1 EDGE=ln⁡(1+ED)+GE⋅ln⁡(2).

At least four endangerment assessments analogous to the IUCN Red List exist for languages: a list by Sutherland [5], a conservation biologist; UNESCO’s atlas of the world’s languages in danger [36]; a database by the Endangered Languages Project (www.endangeredlanguages.com); and the EGIDS scale [37] used by the Ethnologue, an online database of 7000 languages [38]. Given a detailed language phylogeny, it is hence possible to apply techniques from conservation biology to language preservation.

Here, we illustrate how the EDGE framework can be applied to linguistic diversity using a tree of several hundred Austronesian languages built on differences in basic vocabulary (210 words), typically stable through time and resistant to borrowing from other languages. This tree represents one of the largest language families in the world, which probably originated from Taiwan 4000–6000 years ago and then rapidly expanded through islands of the Pacific [39]. The exercise of ranking languages with the EDGE metric can identify languages that are both distinct and threatened, which might be considered important targets for documentation and preservation, if not done already. Although we analyse only a subset of Austronesian language diversity, we present a method that corrects for limited sampling, and show that our results are surprisingly robust to missing languages in the phylogeny.

## 2. Material and methods

### 2.1. Measuring evolutionary distinctiveness

The tree used in this analysis has 1215 tips representing the 1215 living ISO 639-3 Austronesian languages. The tree is a composite of two datasets: a 350-tip tree with branch lengths from Gray *et al.* [40] and, provided in the electronic supplementary material, language classification data by the Ethnologue. Gray *et al.*’s tree is the core dataset. It is based on lexical data from Greenhill *et al.* [41], consisting for each language of 210 basic words thought to be stable over time and resistant to borrowing. Branch lengths in Gray *et al.*’s tree represent the median number of cognate changes undergone on that branch across trees sampled from a Bayesian posterior distribution. Importantly, Gray *et al.* made no assumption that words change at a constant rate over time.

The tree in Gray *et al.* [40] consists of 400 languages chosen based on data availability and to provide ‘a representative sample of each recognized Austronesian subgroup’ [40]. From this set we removed 16 languages that were extinct, not Austronesian or without an ISO 639-3 code from the International Organization for Standardization [42]. We further removed 34 Austronesian dialects that shared an ISO 639-3 code with another language in the tree, always keeping the dialect with the greatest ED. This resulted in a tree with 350 languages (hereafter ‘Gray *et al.*’s tree’).

**Figure 1.**
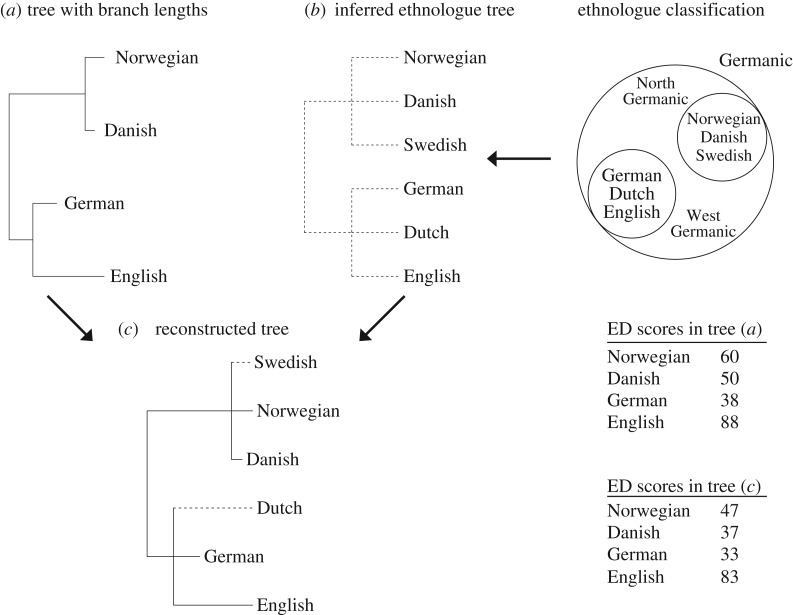
How the Austronesian tree was reconstructed to compute evolutionary distinctiveness more accurately, using Germanic languages as an example. A tree (*a*) of Germanic languages with (here invented) branch lengths can be used to compute evolutionary distinctiveness (ED), but missing languages (Dutch and Swedish) will bias this score. Language classifications into families and subfamilies by the Ethnologue (simplified for illustration) can partially compensate for this bias. It can be used to infer a tree (*b*) with no meaningful branch lengths. Those languages or groups of languages missing from tree (*a*) are imported from tree (*b*) to form a reconstructed tree (*c*). ED, as calculated from tree (*c*), is usually more accurate than when calculated from tree (*a*); see main text. This method does not allow computing ED of languages missing from tree (*a*). In this analysis, we used the Austronesian equivalent of tree (*c*). Details in the electronic supplementary material.

Of the 1215 living Austronesian languages, 71.2% are not represented in Gray *et al.*’s tree, and missing languages may be expected to affect ED scores. To account for this effect, we complemented the phylogeny with language classification data from the Ethnologue, which groups all ISO 639-3 languages into families and subfamilies. The Ethnologue classification for Austronesian languages was converted into a tree with no meaningful branch lengths, and then missing languages were inserted into Gray *et al.*’s tree (figure 1; details in the electronic supplementary material). This resulted in a 1215-tip tree (hereafter the ‘reconstructed full Austronesian tree’) used to calculate ED following the fair proportion method devised by Redding [31]. ED was estimated for only those 350 living ISO 639-3 Austronesian languages present in Gray *et al.*’s tree.

### 2.2. Measuring global endangerment

To measure the probability of extinction, we converted the 10-point EGIDS scale of language endangerment into a Global Endangerment index (GE, table 1). We chose this approach to quantifying GE as it parallels the IUCN Red List [35] and most closely matches to the original EDGE framework published by Isaac *et al.* [18] in which increases of one threat level double the probability of extinction (details and conversion scheme in electronic supplementary material, table S1). Because GE is simply a multiplier in the calculation of EDGE, it would be straightforward to substitute our GE index for alternative estimates of P(extinction), such as those of Sutherland [5], UNESCO’s atlas of the world’s languages in danger [36], or if data are available, estimations of P(extinction) based on the total number of speakers [43]. If uncorrelated with EGIDS, we would expect different endangerment scales to yield different EDGE scores. The EGIDS, however, is the only complete scale for the languages in our sample.

**Table 1.** Definition of global endangerment (GE) scores for language endangerment. GE is a conversion of the EGIDS endangerment scale that parallels Isaac’s conversion of the IUCN Red List, in which increases of one unit in GE represent a doubling in the probability of extinction. The age of youngest users is the most important criterion for the EGIDS scale (details in electronic supplementary material, table S1).

GE	EGIDS endangerment	youngest users	other criteria
4	nearly ext.	grandparents	rarely used
3.5	moribund	grandparents	—
3	shifting	parents	—
2	threatened	children	losing users
1	vigorous	children	stable user base
$\frac{1}{2}$	developing	children	standardized lit.
$\frac{1}{4}$	educational	children	used in schools
$\frac{1}{8}$	wider Comm.	children	used in mass media
$\frac{1}{16}$	provincial	children	local govt. lang.
$\frac{1}{32}$	national	children	national govt. lang.

### 2.3. Effect of missing languages on evolutionary distinctiveness

As mentioned above, missing languages may be expected to affect ED scores, an effect that data from the Ethnologue cannot be expected to correct entirely because of unresolved polytomies. To assess the effect of missing languages, we performed the following sensitivity analyses. Given that 71.2% of the 1215 Austronesian languages are missing in Gray *et al.*’s 350-language tree, we randomly pruned from it 249 languages. This yielded a 101-language tree (hereafter a ‘reduced Gray tree’), lacking 71.2% of the languages in Gray *et al.*’s original tree. We then calculated the *R*^2^ between the ED scores from Gray *et al.*’s tree and the reduced Gray tree. We repeated this process 10 000 times, each time obtaining a new reduced Gray tree by randomly pruning 249 languages. On average, the ED of the reduced Gray tree and that of Gray *et al.*’s tree correlated to an *R*^2^ of 0.78, and to 0.75±0.14, 99% of the time. One may then apply, on the reduced Gray trees, Ethnologue data with the procedure mentioned in §2.1 to partially reconstruct Gray *et al.*’s tree, yielding ‘reconstructed Gray trees’, for which ED scores may be computed for the 101 languages present in the reduced tree. On average, the 101 computable ED scores of each reconstructed Gray tree and those of the corresponding 101 languages in Gray *et al.*’s tree correlated to an *R*^2^ of 0.82, and to 0.78±0.14, 99% of the time.

**Figure 2.**
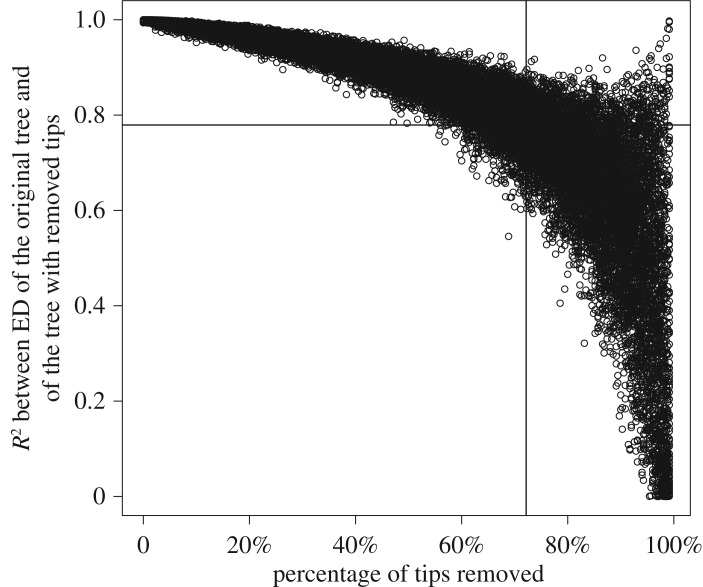
How robust ED is to missing languages. Every one of these 35 000 points represents the *R*^2^ between the ED scores of Gray *et al.*’s tree (350 languages) and the ED scores of one of its subtrees. Each subtree was obtained by randomly removing from Gray *et al.*’s tree a fixed proportion of languages represented on the *x*-axis. Even when 71.2% of tips are removed (vertical line), ED scores correlate well to that of Gray *et al.*’s tree, with *R*^2^=0.78 on average (horizontal line). If subtrees with 101 languages (i.e. with 71.2% of the 350 languages removed) are reconstructed to 350 languageswith Ethnologue data, as detailed in Materials and methods but not depicted here, the average *R*^2^ rises from 0.78 to 0.82. In Gray *et al.*’s tree of 350 languages, 71.2% of the 1215 ISO 639-3 Austronesian languages are missing. We therefore expect that the ED scores of the reconstructed tree used in this analysis are good approximations.

We then generalized the pruning procedure from 249 pruned languages to any number of pruned languages (figure 2, for a similar generalization of the pruning-and-reconstruction procedure; see electronic supplementary material figure S2). It appears that the *R*^2^ between the ED scores of Gray *et al.*’s tree and that of the tree with pruned languages does not decrease linearly with the number of tips removed. The ED scores appear initially resilient. By contrast, if this sensitivity analysis of the pruning procedure is performed not on Gray *et al.*’s tree but on a random tree generated with the ape [44] package of the R statistical language, the *R*^2^ decreases linearly and is on average equal to the percentage of tips left in the reduced tree.

These sensitivity analyses assume that the 1215 languages Gray *et al.* [40] included in their tree were a random subset of all Austronesian languages. Inclusion of languages in the phylogeny is influenced by the availability of data, and there may be bias in favour of well-documented languages, whereas those languages least well-documented might also be among the most endangered. On average, languages present in Gray *et al.*’s tree have an endangerment score of 1.21 and languages absent from the tree, a score of 1.49. Of languages present in Gray *et al.*’s tree, 33% of languages are threatened (GE≥2), whereas this is the case for 41% of excluded languages. Of languages present in Gray *et al.*’s tree, 13% are endangered (GE≥3) meaning that they are only spoken by the parent generation and older, while this is the case for 15% of excluded languages. Of languages present in Gray *et al.*’s tree, 6.9% are moribund (GE≥3.5), meaning that they are only known to the grandparent generation and older. This figure is 7.4% in languages absent from Gray *et al.*’s tree. These figures suggest some bias: less endangered languages are slightly over-represented in the tree.

### 2.4. Calculating the evolutionarily distinct and globally endangered scores

As branch lengths in the language tree were not proportional to time (as is often the case with species trees), an absolute ED score is difficult to interpret. We therefore chose to use the relative ED (ED_R_), computed by dividing all ED scores by the average ED score. By construction, ED_R_ scores have a mean of 1, and a language with an ED_R_ of 2 is twice as distinct as the average language.

The weightings of ED and GE in the original EDGE metric are arbitrary [45]. To give importance to both ED and endangerment (GE), we adapted the EDGE metric given in equation (1.1) by dividing the weight of the GE by 4, its maximum value:

2.1 $$\text{EDGE}=\ln(1+{\mathrm{ED}}_{R})+\frac{1}{4}\cdot \text{GE}\cdot \ln(2).$$

Had we stuck to the original definition of the EDGE, rankings would have been dominated by endangerment scores with little regard to distinctiveness (see Pearse *et al.* [46] for a similar approach). An extension of EDGE, HEDGE (‘heightened’ EDGE), also includes information on the P(extinction) of close relatives, such that an endangered language would be up-weighted if closely related languages were also endangered [47]. While this is a useful approach when setting global conservation priorities, the HEDGE metric is not appropriate in our case due to the large number of missing languages (i.e. we cannot be certain that we were not missing a closely related language that had a very different GE score to a language within our sample).

We ran all analyses with the R statistical language [48] with the following libraries: we used the ade4 [49], ape [44] and phytools [50] R packages to manipulate phylogenetic trees, picante [51] to compute ED, phangorn [52] and geiger [53] to idenfity ancestral nodes, and ggplot2 [54] to generate plots.

## 3. Results

**Figure 3.**
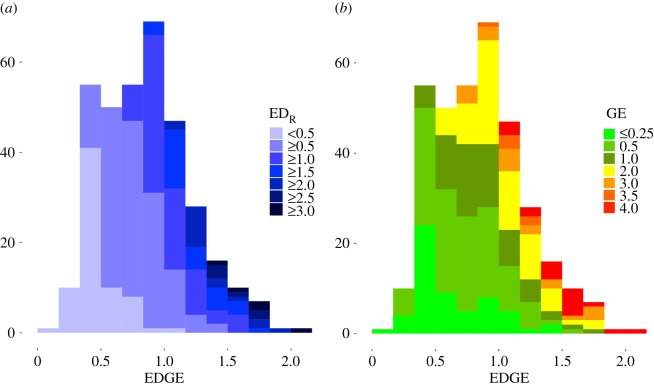
EDGE distribution of the 350 Austronesian languages shaded by theirrelative evolutionary distinctiveness (*a*) and endangermentlevel (*b*).

ED_R_ scores are approximately log-normally distributed (full list in electronic supplementary material), ranging from that of Indonesian (0.15) to those of Kavalan (3.36) and Xârâcùù (Canala) (3.66). ED_R_ scores have a geometric mean of 0.836 and a median of 0.847. Of the 350 languages for which we can measure ED, 113 (32%) are threatened (GE ≥ 2), representing 34% of the total measurable Austronesian ED. EDGE scores are approximately normally distributed but slightly right-skewed (figure 3), and range from 0.15 (Indonesian) to 2.17 (Kavalan, table 2), with an average of 0.786 and a s.d. of 0.35.

**Table 2.** Languages by EDGE score (full list in the electronic supplementary material .csv file).

	language	EDGE	ED_R_	endangerment	GE
1	Kavalan	2.17	3.36	nearly extinct	4
2	Tanibili	1.86	2.21	nearly extinct	4
3	Waropen	1.774	2.50	shifting	3
4	Sengseng	1.765	3.13	threatened	2
5	Magori	1.75	1.88	nearly extinct	4
6	Xârâcùù	1.7133	3.66	vigorous	1
7	Irarutu	1.7130	2.92	threatened	2
⋮					
349	Tuvaluan	0.24	0.25	wider comm.	$\frac{1}{8}$
350	Indonesian	0.15	0.16	national	$\frac{1}{32}$

ED and GE do not appear correlated (*R*^2^=0.008, *p*=0.09), although this could change with different ED and GE metrics. Nonetheless, our choice of GE is just one possible index of language endangerment, and alternative scales or transformations of language threat might reveal the relationship between ED and GE. As discussed above, we assessed the effect of missing languages on ED. We expect a coefficient of determination *R*^2^ of ≈0.82 between their ED scores in the reconstructed full Austronesian tree (the one used in this analysis) and their ED scores in the hypothetical full Austronesian tree (within 0.78±0.14, with 99% probability, details in the electronic supplementary material).

Neither ED_R_ nor EDGE are randomly distributed geographically—the Philippines are a striking example. Of the 350 languages studied here, 53 are of the Philippines (although Filipino itself, also an Austronesian language, is not included). In the Philippines, 48 languages (91%) have ED_R_ below average, and 51 languages (96%) are in vigorous use (GE≤1). The Philippine language with the highest ED_R_, Inabaknon, only ranks 83 out of 350, which is significantly lower than expected by chance (*p*<10^−6^; see the electronic supplementary material). Similarly, the Philippine language with the highest EDGE, Central Tagbanwa, ranks 90 out of 350, again significantly lower than expected by chance (*p*<10^−7^).

As for EDGE scores across other countries, all five French Polynesian languages except Tahitian are endangered, which makes French Polynesia the country with the highest average GE among those countries with more than two languages (avg. GE=2.41, *n*=5). Under the HEDGE framework, these languages would have been given even higher conservation priority. Formosan languages, spoken by indigenous peoples of Taiwan, have the second-highest average GE (1.91, *n*=14). French Polynesian languages, however, have a much lower average ED_R_ (0.44) than Formosan languages (1.91); losing one average Formosan language would reduce the measured Austronesian ED more than losing all four endangered French Polynesian languages. Only New Caledonian languages have higher average ED_R_ (1.93) than Formosan languages, but New Caledonian languages are not as threatened (avg. GE=1.25, *n*=7). High ED and GE make Formosan languages the highest EDGE scoring on average (avg. EDGE=1.38), followed by New Caledonia (1.20).

## 4. Discussion

In linguistics, as in conservation biology, limited resources in conjunction with rapid rates of extinction mean that efforts need to be optimized to maximize the preservation of diversity. Here, we suggest how efforts to preserve linguistic diversity could benefit from approaches used in conservation biology that include both distinctiveness and GE. Applying these types of metrics to languages requires only an endangerment score for each language, and a language tree whose branches reflect linguistic distinctiveness, data that are already available for many languages.

We illustrate the linguistic EDGE on a 350-language Austronesian family tree. Our results reveal striking disparities in the ED among languages, here reflecting a measure of lexical contribution to linguistic diversity. For example, the language with the highest ED, Xârâcùù, contributes 23 times more than the language that contributes least. The six highest ranking EDGE languages (table 2) were Kavalan (ED_R_=3.36, GE=4), Tanibili (ED_R_=2.21, GE=4), Waropen (ED_R_=2.50, GE=3), Sengseng (ED_R_=3.13, GE=2), Magori (ED_R_=1.88, GE=4) and Xârâcùù (ED_R_=3.66, GE=1).

Kavalan is an exceptionally distinct yet nearly extinct language indigenous to Northeastern Taiwan. In 2000, it had 24 speakers [38] and an ethnic population of 1000 living mostly in Eastern Taiwan [55]. It is spoken in only one village, Sinshe, chiefly by elderly speakers. There have been recent efforts to revive it in schools, but without proper funding the village could not train language teachers [56]. Tanibili is one of three highly endangered languages of Utupua in Temotu Province, Solomon Islands, none of which have more than a few hundred speakers and are almost completely undocumented [57]. Waropen and Sengseng are languages of New Guinea spoken by a few thousand people. There are some word lists and other resources for Waropen [58], while there are word lists and a sketch grammar for Sengseng [59,60]. Waropen is no longer spoken by children, and only half of the children of Sengseng users speak it [38]. Magori is a nearly extinct language of Papua New Guinea that had 100 users in 2000 [38]. It is known, however, to have undergone large-scale lexical and structural borrowings from Magi, a Papuan language [61], and because unaccounted borrowings are ignored when computing ED, our estimate of ED_R_ might overestimate the distinctiveness of the language. Xârâcùù is a language of southern New Caledonia spoken by some 6000 people [62], and although not currently endangered, it is considered near threatened.

There are multiple complementary approaches for language preservation. Yet, for largely undocumented languages close to extinction, recording is an essential first step, for if there is no record of a language beyond its current speakers, there will be no reviving it once those speakers are lost. The exercise of ranking languages by both level of endangerment and distinctiveness is useful for identifying global priorities that maximize linguistic diversity. Such prioritization lists, however, can at best only help to inform preservation programs, and do not take into account other factors such as the quantity and quality of existing documentation, the practicality of working in particular regions, or the cultural, social and political contexts unique to each language [63,64]. This is an important observation, as in addition to identifying languages that might be prioritized, we show that neither ED_R_ nor EDGE are randomly distributed geographically. Both linguistic diversity and the drivers of language extinction risk are known to be geographically patterned [65,66], which may offer opportunities to prioritize groups of languages by proximity, leveraging the resources necessary for documentation to multiple languages at once. Similar challenges and opportunities arise in species conservation.

We should be cognisant that our measures of ED reflect only the information that is used to create the tree, and other metrics of ED are available. Any single language tree or metric is unlikely, therefore, to fully capture linguistic diversity. Aside from lexical change (new or modified words for the same things), linguistic change involves semantic change (existing words that shift meanings), phonetic change (change in pronunciation), phonological change (change in the frequency or number of phonemes) and syntactic change (change in syntax). Similarly, different ED metrics can give more or less weight to branches deeper in the tree, and thus capture different language features. These different types of language changes can occur together, either because a change in one aspect of a language provokes changes in the other, or because external factors induce changes on several of these aspects simultaneously. They may not, however, necessarily evolve in synchrony, as changes in one dimension can be independent of changes in another dimension. Our case study is based on lexical diversity, but could well be extended to encompass other dimensions of linguistic diversity [24], and account for uncertainties in the resulting trees. We present here a first attempt at merging threat and distinctiveness for language preservation.

As is the case for the species EDGE program, we anticipate and hope that our approach will be revised and improved through time as alternative phylogenies are constructed, methods are improved and as we refine our knowledge of the status of languages around the globe.

## 5. Conclusion

The EDGE scores presented here provide an illustration of the potential benefits in borrowing methods and theory from one field, here conservation biology, and applying them to another, here language preservation. In other examples, the similarity of language and species trees might find the flow of information reversed [67]. We considered over 350 languages, yet these represent only a subset of Austronesian languages. We show that such missingness has only a limited effect on the ED scores of included languages. Importantly, tree incompleteness never lowers EDGE scores, though it is possible that relative rankings could change. In addition we present a novel method to evaluate robustness of ED measures estimated from incomplete trees, which has utility in biology and linguistics. Languages, however, cannot be assessed if we lack data for them. It is notable that while only 210 words are needed to include additional languages in the phylogeny we used, even these data are missing for the majority of Austronesian languages. Perhaps one of the most pressing priorities, therefore, is to gather the data required to build more inclusive language trees. Large, well-sampled species trees have transformed our understanding of macroevolution [68–71] and helped shape conservation priorities (see Mace *et al.* [72]). The construction of more comprehensive language trees is likely to benefit linguists, anthropologists and historians, as well as biocultural diversity for its own sake.

## Supplementary Material

Perrault_2017_Supplementary_Information.pdf

## Supplementary Material

Perrault_2017_Supplementary_Materials.zip

## Data accessibility

The data supporting this article have been uploaded as part of the electronic supplementary material, and includes the ED_R_, GE, EDGE, ISO 639-3 code and country for each language included in the analysis, as well as each of the phylogenetic trees. ED was calculated based on the Austronesian phylogeny of Gray *et al.* [40] built on data from the Austronesian Basic Vocabulary Database [41], freely available online (https://github.com/D-PLACE/dplace-data/tree/master/phylogenies/gray_et_al2009).
