# Supplementary material for: Tongues on the EDGE: language preservation priorities based on threat and lexical distinctiveness
Source: R Soc Open Sci. 2017 Dec 13;4(12):171218. doi: 10.1098/rsos.171218 (PMC5750020; doi:10.1098/rsos.171218)
Supplement: Perrault_2017_Supplementary_Information.pdf [file rsos171218supp1.pdf]

Tongues on the EDGE: Language preservation priorities based on threat and lexical distinctiveness

Supplementary Information

Nicolas Perrault, Maxwell J. Farrell, T. Jonathan Davies

The trees of this analysis

The following is a list of the kinds of trees used in the analysis. It is included here for easy reference.

There are eight kinds of trees mentioned in the article and in this supplementary information. Four are unique and four are categories of trees that can be generated. The four unique ones are:

1. **Gray *et al.*'s tree** (350 tips with branch lengths). An Austronesian tree produced by Gray *et al.* (2009).[1]
2. **The Ethnologue tree** (1215 tips without branch lengths). This tree is derived from Ethnologue's classification of languages in families and subfamilies.
3. **The hypothetical full Austronesian tree**. (It would have 1215 tips with branch lengths if it existed.) This is the tree we are trying to approximate by stitching clades from the Ethnologue tree onto Gray et al.'s tree.
4. **The reconstructed full Austronesian tree** (1215 tips, 350 of which have branch lengths). This is the tree used in the analysis to compute ED scores. It is an approximation of the hypothetical full Austronesian tree, obtained by stitching clades from the Ethnologue tree onto Gray *et al.*'s tree.

The four kinds of trees that can be generated for sensibility analyses are:

5. **Reduced Gray trees** (101 tips with branch lengths). These are produced by randomly removing 249 tips from Gray *et al.*'s tree in the R statistical language with the `drop.tip()` function of the ape[2] package and the `sample()` function. The number 249 represents 71.2% of the tips in Gray *et al.*'s tree, which is the proportion of ISO 639-3 Austronesian languages missing from Gray et al.'s tree.
6. **Reconstructed Gray trees** (350 tips, 101 of which have branch lengths). These are trees produced by applying on reduced trees the algorithm that, when applied on Gray et al.'s tree, produced the reconstructed full Austronesian tree.
7. **Random trees** (arbitrary number of tips). These were generated with the `rtree()` function of the ape[2] package. Of limited scope in this analysis, they were only used to verify that removing a percentage of tips from a small tree had similar effects on ED scores to removing the same percentage of tips from a bigger tree.
8. **Reduced random trees** Random trees from which a number of tips have been dropped.

Effect of removing a fixed percentage of tips on trees of different sizes

We first investigated whether removing a percentage of tips from a small tree affected ED scores in a similar way to removing that same percentage of tips from a bigger tree.

We generated random trees and removed a percentage of their tips to produce reduced random trees. We found that when this percentage is fixed, the mean R^2 between ED scores of a random tree and those of a reduced random tree does not depend on the size of the random tree. For example, one may start with a random tree of 1000 tips and drop 60% of tips to obtain a reduced random tree of 400 tips. The R^2 between the ED of the random tree (the one with 1000 tips) and the reduced random tree (the one with 400 tips) will average a certain number. If one would rather start with a random tree of 400 tips and drop 60% of tips to obtain a reduced random tree of 160 tips, the R^2 should average the same number. Standard deviations, on the other hand, will be lower when the random tree has more tips.

Effect of missing languages on ED scores

As just mentioned, removing a fixed percentage of tips from a big tree has a similar effect on ED to removing that same percentage on a smaller tree. Gray *et al.*'s tree has 350 tips and so is missing 71.2% of the 1215 Austronesian languages. To study how these missing languages may affect ED scores, one may try and further remove 71.1% of tips, that is 249 tips, from Gray et al.'s tree. One thus obtains a reduced Gray tree with 101 tips. By studying the R^2 between the ED scores of the reduced Gray tree and those of Gray *et al.*'s tree, we obtain an estimate of how close the ED scores of Gray *et al.*'s tree are to the hypothetical ED scores of the hypothetical full Austronesian tree.

On average, the ED scores of a reduced Gray tree and those of Gray *et al.*'s tree correlate to an R^2 of 0.78 with a 99% non-parametric prediction interval (for a single new data point) of 0.75 ± 0.14. This interval was derived from the study of 10,000 reduced Gray trees, each one with 101 tips. The lower bound of the interval is the 51st lowest R^2 of the 10,000 and the upper bound the 51st highest. (The distribution of R^2 was close to the normal distribution but with a left skew, hence this non-parametric prediction interval.) The above results suggest that the ED scores of Gray *et al.*'s tree may be expected to correlate with an R^2 of 0.78 to those of the hypothetical full Austronesian tree, and within 0.75 ± 0.14 with 99% confidence at the least. In fact, we would expect the 99% prediction interval to be much tighter about the central value of 0.75, because this prediction interval was calculated on Gray *et al.*'s tree rather than on the hypothetical full Austronesian tree and the standard deviation usually decreases when the full tree is larger. This means that the ED of Gray *et al.*'s tree very probably correlates to the ED of the hypothetical full Austronesian tree with an R^2 in that prediction interval.

The reconstruction algorithm

The following paragraphs suggest a way of rising the expected R^2 from 0.78 to 0.82. The Ethnologue classifies languages in families and subfamilies, just like a species is classified into a domain, a kingdom, a phylum, a class, an order, a family, and a genus. With permission, we scraped the classification off the site, and used the `as.phylo.formula()` from the ape package[2] of the R statistical language[3] to convert this taxonomic classification of languages into what we call the "Ethnologue tree", one with 1215 languages but no meaningful branch lengths.

Although we need meaningful branch lengths to compute the EDGE, we only need them for languages in whose EDGE we are interested. Gray *et al.*'s tree has meaningful branch lengths but the Ethnologue tree does not. We therefore stitched from the Ethnologue tree and into Gray *et al.*'s tree those clades of languages missing in Gray *et al.*'s tree. We call the resulting tree the "reconstructed full Austronesian tree". The algorithm to perform the reconstruction is as follows (see also Figure 1):

1. In the Ethnologue tree, highlight every (largest) monophyletic group of tips that are not in Gray *et al.*'s tree. Call them "absent clades".

2. For each absent clade, find the single language or group of languages in Gray *et al.*'s tree that is its closest relative in the Ethnologue tree.

3. (a) If it is a *single language*: Find in Gray *et al.*'s tree its most recent ancestor node, that is, the node that directly subtends this language. Call this the "MRA node".

   (b) If it is a *group of languages*: Find in Gray *et al.*'s tree the most recent common ancestor node of these languages. Call this "the MRCA node".

4. Stitch the absent clade from the Ethnologue tree at the MRA or MRCA node in Gray *et al.*'s tree. Repeat steps 2. to 4. for every remaining absent clade.

The resulting tree has all 1215 Austronesian languages. To test how accurately it allows measuring ED scores, we used this algorithm to reconstruct (from 101 tips to 350 tips) the 10,000 reduced Gray trees mentioned above. Reconstructing 10,000 trees from 101 to 350 tips took 11 days of computer time (on a laptop). Whereas the ED scores of the reduced Gray trees correlated with those of Gray *et al.*'s tree to an average R^2 of 0.78 (0.75 ± 0.14, 99% of the time), the ED scores of the reconstructed Gray trees correlated with those of Gray *et al.*'s tree to an average R^2 of 0.82 (0.78 ± 0.14, 99% of the time).

In terms of ED, reconstructed Gray trees were almost always closer to Gray *et al.*'s tree than were the respective reduced Gray trees. The reconstructed trees did better than the reduced trees in 9822 cases out of 10,000. This suggests that when 71.2% of tips are removed from Gray *et al.*'s true, the reconstruction algorithm betters the estimate of ED scores 98.20 ± 0.35% of the time (99% conf., binomial exact test). In the remaining 178 cases, the algorithm worsened the estimate, though typically not by much, as shown by the following analysis of the betterment.

We measure the "betterment" of a reconstruction method by the proportion of uncertainty it removes from the reduced tree. In this context, uncertainty is defined as $1 - R^2$. Because the ED scores of reduced trees correlate to those of Gray *et al.*'s tree to an average R^2 of 0.78, reduced trees may be said to have an average ED uncertainty of 0.22 (= 1 − 0.78). The ED scores of reconstructed trees likewise correlate to those of Gray *et al.*'s tree to an average R^2 of 0.82. Reconstructed trees may therefore be said to have an average ED uncertainty of 0.18 (= 1 − 0.82). We then say that the reconstruction algorithm betters the ED estimate of the reduced trees by 18.2% because, of the reduced trees' ED uncertainty of 0.22, 18.2% has been removed by the reconstruction:

$$0.22 \cdot (100\% - 18.2\%) \; = \; 0.18$$

In general the betterment may be computed as follows:

$$\frac{\text{ED uncertainty in reduced tree} - \text{ED uncertainty in reconstructed tree}}{\text{ED uncertainty in reduced tree}}$$

$$= \frac{(1 - \mathrm{R}^2_{\text{reduced}}) - (1 - \mathrm{R}^2_{\text{reconstructed}})}{1 - \mathrm{R}^2_{\text{reduced}}}$$

$$= 1 - \frac{1 - \mathrm{R}^2_{\text{reconstructed}}}{1 - \mathrm{R}^2_{\text{reduced}}}$$

The following are prediction intervals for the betterment of the reconstruction method applied on reduced Gray trees (with 101 tips). These intervals make no assumption regarding the statistical distribution of the betterment, but were observed over 10,000 trial reconstructions.

[+10.5%, +24.2%]	50% of the time
[+6.2%, +30.3%]	80% of the time
[+1.2%, +37.1%]	95% of the time
[−4.6%, +43.4%]	99% of the time
[−12.5%, +50.7%]	99.9% of the time

For example, 50% of the time, the reconstruction algorithm betters the estimated ED scores of the reduced Gray tree by more than 10.5% and by less than 24.2%. The above prediction intervals suggest that we can use the reconstruction algorithm to obtain a reconstructed full Austronesian tree, and that its ED scores will probably be closer to those of the hypothetical full Austronesian tree than are the ED scores from Gray *et al.*'s tree. Indeed, we predict that the ED scores of the reconstructed full Austronesian tree used in this analysis will be better than those of Gray *et al.*'s tree by a factor given in the above prediction intervals.

In the sensibility analysis just detailed, 71.2% of tips were removed from Gray *et al.*'s tree. We then generalised this analysis by allowing for an arbitrary number of removed tips. We found that the algorithm works well regardless of the number of tips removed (Figure 2). In general, the betterment is greater when fewer tips are removed. When 1% of tips are removed, the betterment averages 24%. This means that, should the $\mathrm{R}^2_{\text{reduced}}$[1] be of 0.99900 when 1% of tips are removed, the $\mathrm{R}^2_{\text{reconstructed}}$ is predicted to average 0.99924. When 60% of tips are removed, the betterment averages 20%. When, as above, 71.2% of tips are removed the betterment

[1] $\mathrm{R}^2_{\text{reduced}}$ is the R^2 between the ED scores of the reduced Gray tree and those of Gray *et al.*'s tree. $\mathrm{R}^2_{\text{reconstructed}}$ is the R^2 between the ED scores of the reconstructed Gray tree and those of Gray *et al.*'s tree.

averages 18.2%. When 90% of tips are removed, the betterment averages 12%. In absolute terms, however, the reconstruction algorithm increases the R^2 more on trees with a high number of removed tips, because when few tips are removed, there is not much uncertainty to remove in the first place.

Deriving p-values for languages of the Philippines

The main text states that “the Philippine language with the highest ED_R, Inabaknon, only ranks 83rd out of 350, which is significantly lower than expected by chance ($p < 10^{-6}$).”

Let *Rank* be the ED rank of the Philippine language with highest ED. The p-value is the probability that the highest Philippine ED be of rank greater or equal to 83 under the null hypothesis that ED scores are randomly distributed geographically. That is, if P_0 denotes the probability under the null hypothesis,

$$
\begin{aligned}
p &= \mathrm{P}_0(Rank \geq 83) \\
&= \mathrm{P}_0(Rank \notin \{1, 2, ..., 82\}) \\
&= \mathrm{P}_0(Rank \neq 1) \cdot \mathrm{P}_0(Rank \neq 2 | Rank \neq 1) \times \mathrm{P}_0(Rank \neq 3 | Rank \notin \{1,2\}) \times \cdots \times \mathrm{P}_0(Rank \neq 82 | Rank \notin \{1, 2, ..., 81\}) \\
&= \Big(1 - \mathrm{P}_0(Rank = 1)\Big) \times \Big(1 - \mathrm{P}_0(Rank = 2 | Rank \neq 1)\Big) \times \cdots \times \Big(1 - \mathrm{P}_0(Rank = 82 | Rank \notin \{1, 2, ..., 81\})\Big) \\
&= \left(1 - \frac{53}{350}\right) \times \left(1 - \frac{53}{350 - 1}\right) \times \cdots \times \left(1 - \frac{53}{350 - 81}\right) \qquad \text{(There are 53 Philippine languages)} \\
&= \prod_{i=0}^{81} \left(1 - \frac{53}{350 - i}\right) \\
&\approx 1.83182 \times 10^{-7} < 10^{-6}
\end{aligned}
$$

The main text continues, stating that “the Philippine language with the highest EDGE, Central Tagbanwa, ranks 90th out of 350, again significantly lower than expected by chance ($p < 10^{-7}$).” Let *Rank* be the EDGE rank of the Philippine language with highest EDGE. The p-value is obtained as above with

$$
\begin{aligned}
p &= \mathrm{P}_0(Rank \geq 90) \\
&= \prod_{i=0}^{88} \left(1 - \frac{53}{350 - i}\right) \\
&\approx 3.84118 \times 10^{-8} < 10^{-7}
\end{aligned}
$$

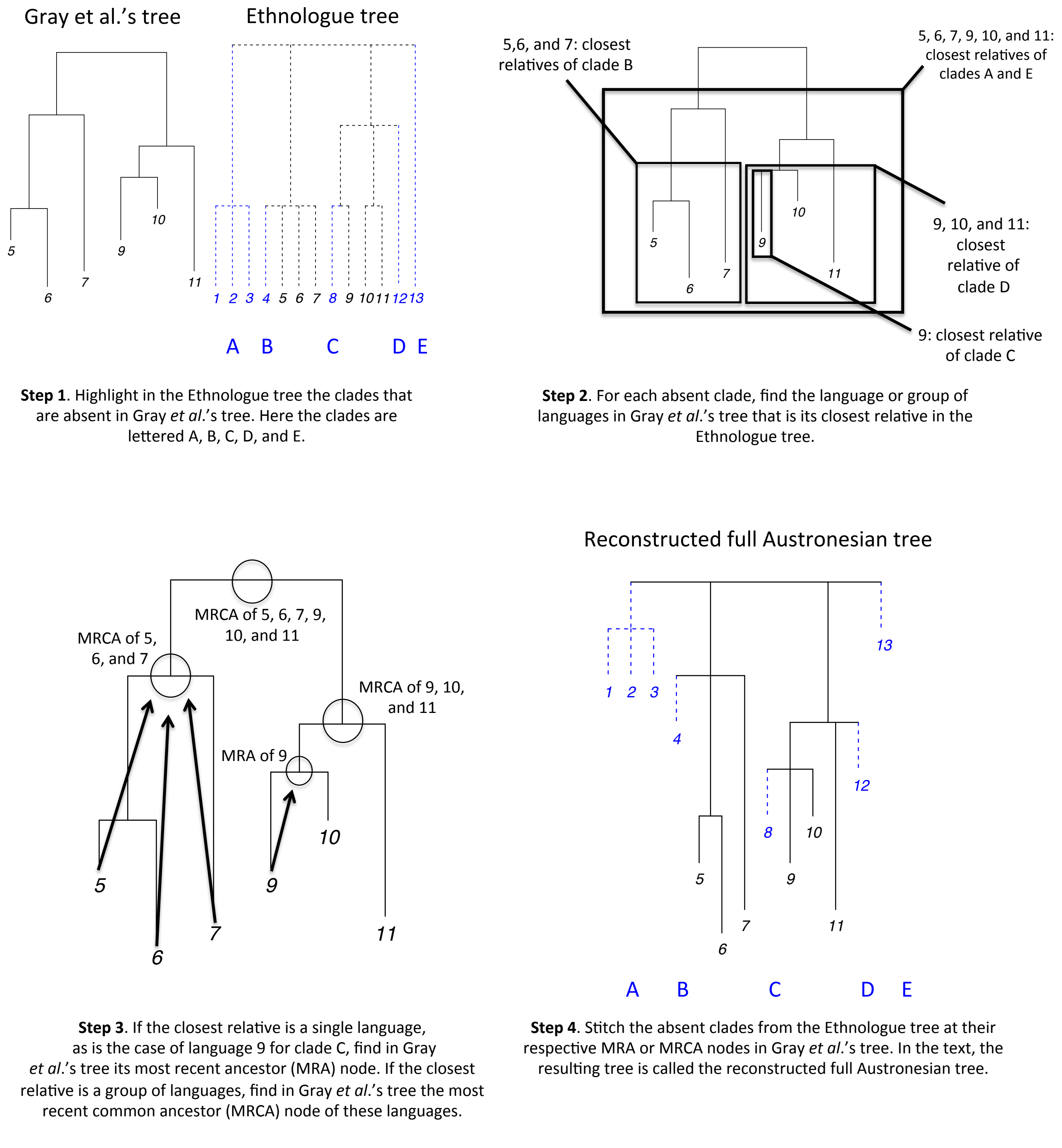

Step 1. Highlight in the Ethnologue tree the clades that are absent in Gray *et al.*'s tree. Here the clades are lettered A, B, C, D, and E.

Step 2. For each absent clade, find the language or group of languages in Gray *et al.*'s tree that is its closest relative in the Ethnologue tree.

Step 3. If the closest relative is a single language, as is the case of language 9 for clade C, find in Gray *et al.*'s tree its most recent ancestor (MRA) node. If the closest relative is a group of languages, find in Gray *et al.*'s tree the most recent common ancestor (MRCA) node of these languages.

Step 4. Stitch the absent clades from the Ethnologue tree at their respective MRA or MRCA nodes in Gray *et al.*'s tree. In the text, the resulting tree is called the reconstructed full Austronesian tree.

Figure 1: The reconstruction algorithm. Notice that in this example, Gray *et al.*'s tree and the Ethnologue tree have different typologies for languages 9, 10, and 11.

Table 1: Ethnologue's EGIDS scale of language endangerment, with our IUCN Red List (GE) equivalents

	EGIDS	Definition	GE	IUCN Red List equivalent
0	International	The language is widely used between nations in trade, knowledge exchange, and international policy.	0	Least Concern
1	National	The language is used in education, work, mass media, and government at the national level.	$1/32$	
2	Provincial	The language is used in education, work, mass media, and government within major administrative subdivisions of a nation.	$1/16$	
3	Wider Comm.	The language is used in work and mass media without official status to transcend language differences across a region.	$1/8$	
4	Educational	The language is in vigorous use, with standardization and literature being sustained through a widespread system of institutionally supported education.	$1/4$	
5	Developing	The language is in vigorous use, with literature in a standardized form being used by some though this is not yet widespread or sustainable.	$1/2$	
6a	Vigorous	The language is used for face-to-face communication by all generations and the situation is sustainable.	1	Near Threatened
6b	Threatened	The language is used for face-to-face communication within all generations, but it is losing users.	2	Vulnerable
7	Shifting	The child-bearing generation can use the language among themselves, but it is not being transmitted to children.	3	Endangered
8a	Moribund	The only remaining active users of the language are members of the grandparent generation and older.	3.5	
8b	Nearly Ext.	The only remaining users of the language are members of the grandparent generation or older who have little opportunity to use the language.	4	Critically Endangered
9	Dormant	The language serves as a reminder of heritage identity for an ethnic community, but no one has more than symbolic proficiency.	–	Extinct
10	Extinct	The language is no longer used and no one retains a sense of ethnic identity associated with the language.	–	

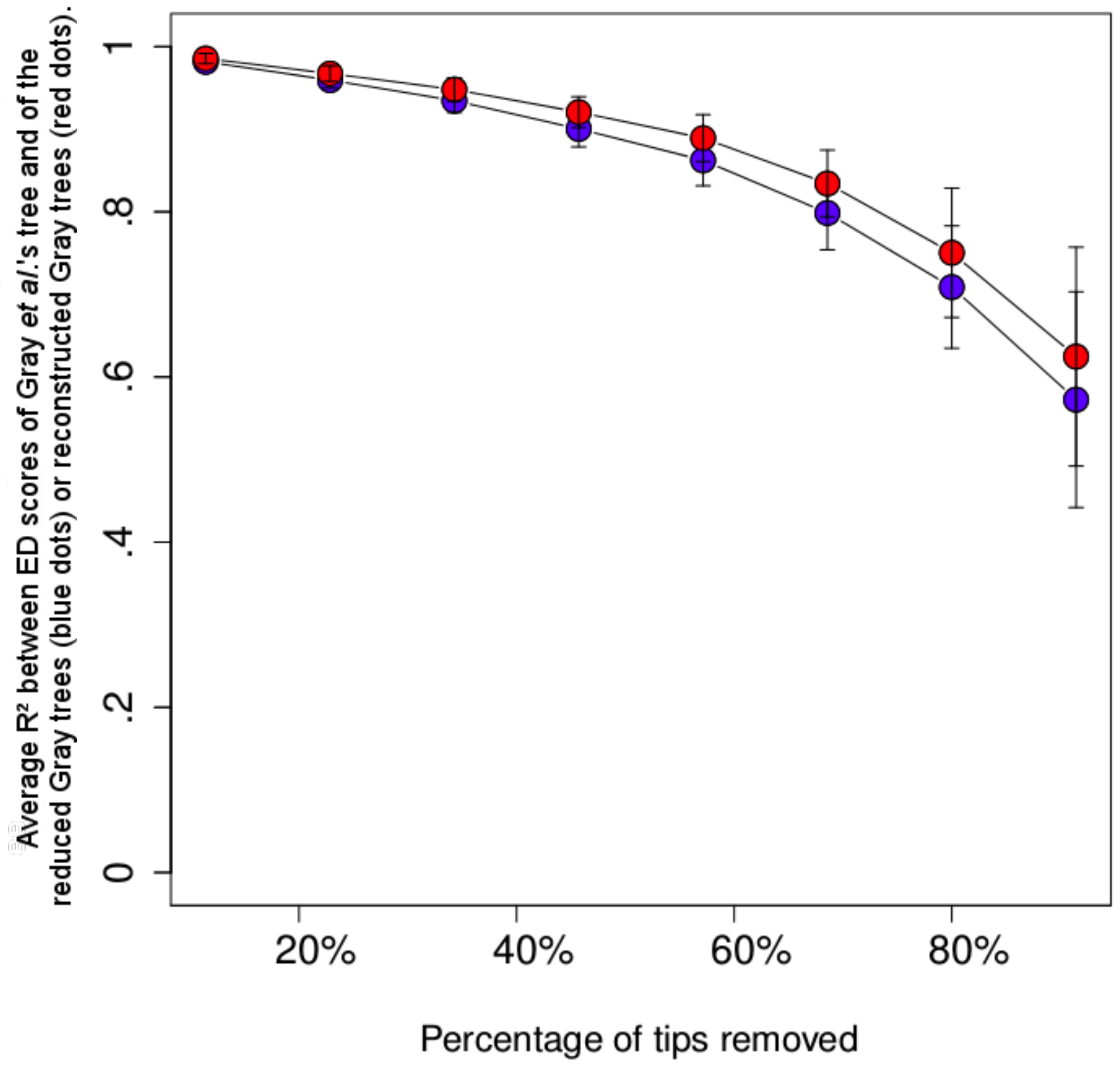

Figure 2: Effect of the reconstruction algorithm. Blue dots represent the average R^2 between the ED scores of the reduced Gray trees and those of Gray *et al.*'s tree. Red dots represent the average R^2 between the ED scores of the reconstructed Gray trees and those of Gray *et al.*'s tree. Error bars are of one standard error. For any number of removed tips, the mean of the red dots is always significantly higher than that of the blue dots.

References

[1] Russell D. Gray, Alexei J. Drummond, and Simon J. Greenhill. Language phylogenies reveal expansion pulses and pauses in Pacific settlement. *Science*, 323(5913):479–483, 2009.

[2] Emmanuel Paradis, Julien Claude, and Korbinian Strimmer. Ape: Analyses of phylogenetics and evolution in R language. *Bioinformatics*, 20(2):289–290, 2004.

[3] R Core Team. *R: A Language and Environment for Statistical Computing*. R Foundation for Statistical Computing, Vienna, Austria, 2011.
